# A Pilot Study of the Effectiveness and Safety of Subcutaneous Infliximab in Chronic Inflammatory Pouch Conditions: The St. Mark’s Experience

**DOI:** 10.3390/jcm15052053

**Published:** 2026-03-08

**Authors:** Itai Ghersin, Orestis Argyriou, Kapil Sahnan, Janindra Warusavitarne, Ailsa L. Hart

**Affiliations:** 1 Department of Gastroenterology, St Mark’s National Bowel Hospital & Academic Institute, London NW10 7NS, UK; ailsa.hart@nhs.net; 2 Department of Colorectal Surgery, St Mark’s National Bowel Hospital & Academic Institute, London NW10 7NS, UK; orestis.argyriou@nhs.net (O.A.); kapil.sahnan@nhs.net (K.S.); j.warusavitarne@nhs.net (J.W.); 3 Department of Surgery and Cancer, Imperial College London, London SW7 2AZ, UK; 4 Department of Metabolism, Digestion and Reproduction, Imperial College London, London SW7 2AZ, UK

**Keywords:** infliximab, pouchitis, proctocolectomy, restorative, tumor necrosis factor inhibitors

## Abstract

**Background/Objectives**: Infliximab (IFX) is commonly used in chronic inflammatory conditions of the ileo-anal pouch. A subcutaneous (SC) formulation has been developed, with studies in inflammatory bowel disease (IBD) patients showing that switching from intravenous (IV) to SC IFX is safe with a low risk of relapse. However, so far, it has not been specifically investigated in chronic inflammatory pouch conditions. The aim of our study was to evaluate the effectiveness and safety of SC IFX in patients with chronic inflammatory pouch conditions. **Methods**: This was an observational retrospective study. We included patients with chronic inflammatory pouch conditions, initially treated with IV IFX and subsequently switched to SC IFX, who had a follow-up of at least 1 year. The primary outcome was SC IFX treatment persistence, defined as continuation of SC IFX throughout the study period. The secondary outcome was pouch failure, defined by the need for a defunctioning ileostomy or pouch excision. **Results**: A total of seven patients were included. The mean age was 50.6 years. The average follow-up length was 101.3 months (range 70.4–132.6 months). All seven patients continued SC IFX throughout the study period. No patient experienced pouch failure. The median IFX serum concentration was 18.1 mg/L. There were no cases of serious infections or malignancy. **Conclusions**: Switching clinically stable patients with chronic inflammatory pouch conditions from IV to SC IFX formulation appears feasible. These findings warrant confirmation in larger patient cohorts.

## 1. Introduction

Despite advancements in medical therapies for ulcerative colitis (UC), about 15% of patients will require a colectomy 10 years after their diagnosis. Subsequent surgical management options include restorative options such as an Ileoanal Pouch Anastomosis (IPAA) or an Ileorectal Anastomosis. Patients can also elect to keep their ileostomy [1]. Despite its effectiveness, IPAA is associated with a range of postoperative complications, most notably pouchitis. Acute pouchitis occurs in approximately 60–80% of patients following surgery [2,3], and among these individuals, 10–15% subsequently develop chronic pouchitis [4,5].

In addition to the above-mentioned complications, some individuals experience Crohn’s disease (CD)-like inflammatory manifestations involving the pouch, including deep ulcerations, fistula formation, and prepouch ileitis (PPI) [6]. The underlying causes and pathogenic mechanisms driving these chronic inflammatory pouch disorders remain poorly understood [7].

While most patients with chronic pouchitis improve with antibiotic therapy [8,9,10], a subset develops antibiotic dependency, requiring extended or recurrent treatment [11]. Furthermore, a minority progress to antibiotic-refractory disease, often necessitating a transition to biologic therapy [12]. To date, vedolizumab remains the only biologic formally evaluated in a randomized controlled trial for this indication [13]. However, anti-tumor necrosis factor (anti-TNF) agents like infliximab (IFX) are frequently employed off-label to manage chronic inflammatory pouch conditions [10].

Existing evidence for the use of IFX in chronic inflammatory conditions of the ileal pouch is limited to small, retrospective, single-centre cohorts with restricted follow-up [14,15,16,17]. While these data suggest that IFX can elicit short-term clinical responses in many patients [18], larger, high-quality studies are essential to validate these outcomes and establish definitive clinical guidelines.

Our group’s previous study of 34 patients with chronic inflammatory pouch disorders found that first-line IFX treatment was effective in roughly half of the cohort after one year. Notably, the majority of patients who did not respond to IFX were still able to avoid a permanent ileostomy by transitioning to alternative biologic therapies. It should be noted that all patients involved in this study received the IV formulation of IFX [19].

The recent development of a subcutaneous (SC) formulation of IFX represents a paradigm shift in inflammatory bowel disease (IBD) management. In patients with CD and UC, the SC formulation has demonstrated non-inferiority to IV administration regarding clinical efficacy and pharmacokinetic profiles. Switching from IV to SC IFX is associated with a low risk of relapse [20,21,22]. It also often results in higher trough levels, due to the steady-state kinetics of SC delivery compared to the “peak and trough” cycle of IV infusions [23].

Despite these advancements, the specific application of SC IFX in patients with chronic inflammatory pouch conditions remains underexplored. This study aims to evaluate the effectiveness and safety of SC IFX in patients with chronic inflammatory pouch conditions.

## 2. Materials and Methods

### 2.1. Study Design and Setting

This was a retrospective single-centre observational study conducted at St. Mark’s, the National Bowel Hospital, London, UK.

### 2.2. Infliximab Switching from IV to SC

Within our IBD service, a standardised transition protocol is offered to patients maintaining clinical remission on IV IFX. This elective switch to SC administration is extended to all patients in clinical remission, including those with an IPAA. In the context of IPAA patients, clinical remission is defined as a PDAI score < 7 [24]. The transition process is governed by a shared decision-making model, wherein the choice to adopt SC maintenance or continue the established IV regimen is determined by patient preference following a clinical consultation.

### 2.3. Infliximab SC Dosing Regimen

SC IFX is routinely administered at a dosage of 120 mg every other week. In patients with low trough serum IFX concentrations, without anti-drug antibodies, we offer to increase the treatment frequency to weekly.

### 2.4. Patient Selection

Inclusion criteria were:

- Undergone a subtotal colectomy for UC with subsequent IPAA formation.
- Endoscopic evidence of a chronic inflammatory pouch condition, with histological confirmation of inflammation.
- Documented use of antibiotics for pouch-related symptoms prior to starting IFX.
- Diagnosis of chronic antibiotic-refractory pouchitis (CARP).
- Prior treatment with ≥1 IV dose of IFX indicated for chronic pouch inflammation following colectomy.
- Subsequently switched from IV IFX to SC IFX.
- Minimum follow-up of one year after switch to SC IFX.
- One of the following reported outcomes within 2 weeks of their final SC IFX dose:
  o Pouch failure, defined as the need for a defunctioning ileostomy, with or without pouch excision.
  o Transition to a different advanced IBD therapy (within or outside the anti-TNF class) following treatment failure, characterised by either primary non-response or secondary loss of response.
  o Cessation of SC IFX therapy due to antibody development, allergic reactions, or treatment-related adverse events and safety issues.
  o Documented instruction to continue SC IFX at the most recent clinical encounter.

Exclusion criteria:

- IPAA performed for indications other than ulcerative colitis, specifically familial adenomatous polyposis or CD.
- Incomplete data due to loss to follow-up.

Diagnostic criteria for pouchitis relied on a PDAI score ≥ 7 recorded within one year of starting IFX [24]. For pre-pouch ileitis (PPI), diagnosis was based on endoscopic evidence of inflammation at the pouch inlet, specifically involving signs such as mucosal oedema, ulceration, or contact bleeding.

### 2.5. Clinic Follow-Up Regimen

Patients with an IPAA receiving IFX maintenance therapy undergo structured follow-up at six-month intervals. This longitudinal follow-up encompasses a standardised clinical assessment alongside proactive therapeutic drug monitoring, including the quantification of serum IFX trough concentrations and anti-drug antibody titers. While endoscopic evaluation is not performed on a fixed temporal schedule, pouchoscopy is utilized at the treating gastroenterologist’s discretion.

### 2.6. Outcomes

Our study evaluated two key clinical outcomes:

**Primary Outcome:** SC IFX treatment persistence, defined as medication continuation to the end of the study period.

**Secondary Outcome:** Pouch failure, defined as the requirement for a defunctioning ileostomy (with or without pouch excision).

### 2.7. Ethics

The study protocol was approved by the Health Research Authority (HRA) and conducted in strict accordance with the ethical principles outlined in the Declaration of Helsinki.

## 3. Results

### 3.1. Patient Baseline Characteristics

Seven patients met the inclusion criteria. The mean age of the cohort was 50.6 years, with an average overall follow-up length of 101.3 months (range 70.4–132.6), and an average follow-up length after switching to SC IFX of 29.5 months (range 13.2–43.6).

None of the patients had IFX treatment prior to colectomy. Two of the patients were smokers (Table 1). Individual patient data is presented in Supplementary Table S1.

The most common indication for IFX treatment was chronic pouchitis with concomitant pre-pouch ileitis (5/7 patients). Chronic pouchitis without pre-pouch ileitis was the treatment indication in two patients (25.5% of the cohort). Cuffitis was present in two patients, and two patients had pouch-related fistulae.

In six patients, IFX was their first-line biologic treatment post-colectomy. One patient received Vedolizumab prior to commencing IFX, with a primary non-response to Vedolizumab.

Four patients received IFX in combination with Azathioprine, and three received it as monotherapy. All four patients receiving azathioprine continued the treatment throughout the study period.

All patients were clinically stable at the time of the switch from IV to SC IFX, with an average PDAI of 3.6 (range 2–5).

### 3.2. Treatment Outcomes

All seven patients (100%) successfully continued SC IFX throughout the study period, meeting the primary outcome of treatment persistence. No patients required a switch to another biologic class or an in-class switch due to loss of response.

Post-treatment endoscopic and histological data were available for six of the seven patients. One patient was offered an endoscopic assessment of his response to treatment but declined. Due to the retrospective nature of the study, the timing of these assessments was not standardized but occurred at the discretion of the treating gastroenterologist. At follow-up, three patients achieved combined endoscopic and histological remission, while the remaining three had active inflammation on endoscopy and histology. It is worth noting that despite persistent endoscopic and histologic inflammation, these three patients remained clinically stable. Consequently, their gastroenterologists continued IFX therapy.

There were no instances of pouch failure, meaning all patients avoided the need for a defunctioning ileostomy or pouch excision.

### 3.3. Pharmacokinetic Monitoring

Pharmacokinetic monitoring revealed a median IFX serum concentration of 18.1 mg/L (range 11.2–27.9), suggesting robust drug exposure following the switch. As a result, dose escalations or interval adjustments of SC IFX were not made. Six out of seven patients did not develop anti-IFX antibodies. Interestingly, one patient developed low-level anti-IFX antibodies (21 AU/mL) while also having a very high IFX serum concentration (27.9 mg/L). The case was discussed in our multi-disciplinary team (MDT), and the antibodies were thought to be non-neutralising antibodies. A decision was made to continue SC IFX, and no adverse outcomes were subsequently observed.

### 3.4. Safety

The switch was well-tolerated. There were no cases of serious infections, malignancies, or treatment-discontinuing adverse events reported during the follow-up.

## 4. Discussion

To the best of our knowledge, this is the first study to describe SC IFX treatment in patients with chronic inflammatory pouch conditions. Our findings demonstrate that switching from IV to SC IFX might be a reasonable strategy for patients with chronic inflammatory pouch conditions. In our cohort, all patients maintained treatment persistence, and notably, none experienced pouch failure, a critical outcome given the profound morbidity and reduced quality of life associated with it [25,26].

The efficacy of SC IFX in our study is in line with the broader IBD literature, such as the REMSWITCH study [27], which established that switching stable IBD patients to SC IFX does not compromise clinical remission or biochemical control. A standout finding in our study was the high median serum concentration. This high trough level is consistent with observations that SC administration provides more stable drug concentrations and higher trough drug levels compared to the “peak-and-trough” fluctuations inherent in IV dosing [22]. Higher trough levels have been consistently associated with improved long-term outcomes and mucosal healing in IBD [28]. By avoiding the low troughs seen with IV infusions, SC delivery may reduce the risk of immunogenicity and subsequent secondary loss of response, which is often driven by sub-therapeutic drug exposure [22,29,30,31]. A recent study examining long-term anti-TNF treatment outcomes in patients with chronic inflammatory pouch conditions found that a significant proportion of patients lost response to anti-TNFs due to immunogenicity [32].

Beyond individual clinical outcomes, the transition to SC IFX offers significant structural benefits for healthcare systems.

Infusion unit capacity is a growing concern among IBD centres [33,34]. IV Infliximab administration is resource-intensive, requiring physical bed space, specialized nursing oversight, and several hours for infusion and post-administration monitoring. Transitioning stable patients to home-based SC injections can alleviate pressure on over-burdened infusion units, freeing capacity for patients requiring complex IV-only therapies or those undergoing biologic induction [35,36].

From an economic perspective, SC IFX is often more cost-effective when evaluating the total cost of care. While drug acquisition costs for SC formulations can be slightly higher, these are largely offset by the elimination of non-drug costs, including nursing hours, facility fees, and consumables [37]. Real-world data suggests that switching to SC formulations can be cost-neutral or even cost-saving for national health services [36].

SC IFX also has potential benefits from a patient autonomy and quality of life perspective. The convenience of self-administration allows patients to manage their condition around their professional and personal lives, significantly reducing “hidden costs” such as travel expenses and time taken off work. Studies have shown high patient acceptance and improved treatment satisfaction following the switch to SC IFX, largely due to this increased flexibility [20,38].

The safety profile observed in our cohort, specifically the absence of serious infections or malignancies, reassures clinicians that the higher trough levels associated with SC delivery do not translate to increased adverse events in the pouch population. This mirrors safety data from larger IBD cohorts and other immune-mediated inflammatory diseases, where SC IFX has shown a comparable adverse event profile to IV therapy [20,21,27]. Furthermore, the steady-state kinetics of SC administration may actually lower the risk of developing anti-drug antibodies, which are frequently triggered by the low trough levels associated with traditional IV dosing intervals, and can cause significant adverse events [20,39,40,41].

The shift toward SC delivery as a standard of care is also gaining momentum with other biologic classes, most notably anti-integrin therapies. The SC formulation of vedolizumab has been robustly validated in the broader IBD landscape through the VISIBLE clinical trial program. Specifically, VISIBLE 1 [42] and VISIBLE 2 [43] demonstrated that SC vedolizumab is highly effective as a maintenance therapy for both UC and CD, providing clinical remission rates and safety profiles comparable to the established IV regimen. Similar to the pharmacokinetic advantages seen with SC IFX, SC vedolizumab results in higher trough concentrations and more stable serum drug levels, which may be advantageous in maintaining long-term mucosal healing [44].

However, despite this strong evidence base in conventional IBD, there remains a striking paucity of data regarding the use of SC vedolizumab specifically for chronic inflammatory pouch conditions. While the EARNEST trial provided landmark randomized controlled evidence for the efficacy of IV vedolizumab in chronic pouchitis [13], this success has not yet been formally replicated or described using the SC formulation in this unique patient population. Given that the ileal pouch represents a distinct anatomical and physiological environment, clinical data is urgently needed to confirm if the pharmacokinetic benefits of SC vedolizumab translate into positive clinical outcomes for chronic inflammatory pouch conditions, as we have observed with SC IFX in the present study.

In contrast to anti-TNF and anti-integrin agents, there is emerging but limited evidence for the use of other SC biologics such as ustekinumab in chronic inflammatory pouch conditions. Ustekinumab, an IL-12/23 p40 antagonist approved for CD [45] and UC [46], has been explored in retrospective cohorts and systematic reviews for its utility in CD of the pouch and chronic pouchitis. In a multicentre cohort of patients with CD of the pouch and chronic pouchitis, ustekinumab demonstrated clinical response and endoscopic improvement, supporting its consideration as a therapeutic option in refractory cases where anti-TNFs and vedolizumab have failed [47]. Moreover, a prospective open-label study evaluating ustekinumab specifically in chronic pouchitis showed meaningful reductions in disease activity indices, suggesting potential efficacy in this difficult-to-treat subgroup [48]. Despite these signals, the overall evidence remains limited and largely based on small, observational, or retrospective studies, underscoring the need for prospective controlled trials to better delineate the role of ustekinumab in these conditions.

Evidence for other IL-23-targeted SC biologics such as risankizumab, mirikizumab, and guselkumab in chronic inflammatory pouch disorders is even more sparse. Risankizumab has recently been reported in a prospective multicenter observational study to induce clinical remission in a significant proportion of patients with CD of the pouch, providing the first peer-reviewed clinical data for this agent in the pouch population and highlighting a potential role for novel IL-23 inhibition beyond Ustekinumab [49]. However, cases and small series remain essentially anecdotal, and larger cohort studies are required to validate these findings. Mirikizumab has been described in the literature in isolated case reports demonstrating successful induction in chronic antibiotic-refractory pouchitis, but formal clinical trial data are not yet available [50]. Similarly, although guselkumab was recently established in both UC [51] and CD [52], including treatment protocols using SC induction instead of IV induction [53,54], there are currently no dedicated clinical studies of guselkumab in chronic pouch conditions, reflecting a significant gap in the evidence. The lack of robust, controlled data for these newer agents highlights an urgent need for research to determine whether the pharmacologic advantages seen in traditional IBD populations translate into meaningful clinical and mucosal healing outcomes in chronic inflammatory pouch disorders [55].

We acknowledge several limitations inherent in our study design that warrant careful consideration when interpreting our findings. First, the small sample size of our cohort is a significant limitation. While chronic inflammatory pouch conditions represent a rare and heterogeneous phenotype, making the recruitment of large cohorts inherently challenging, the limited number of participants reduces the statistical power of our analysis. This small number increases the risk of Type II errors, potentially obscuring smaller but clinically significant differences, and limits our ability to perform robust subgroup analyses or multivariate adjustments for potential confounders. The small sample size of our cohort also precluded a robust statistical correlation analysis between serum IFX concentrations, the development of anti-drug antibodies, and clinical efficacy.

In addition, the retrospective nature of the study introduced further methodological constraints, most notably the lack of a dedicated control group. Without a comparative arm, we cannot definitively attribute the observed clinical stability solely to the SC switch. The absence of a control group makes it difficult to account for the natural history of the disease or the “placebo effect” associated with a change in delivery method. Consequently, our results should be viewed as an observational signal of feasibility rather than a definitive proof of comparative efficacy.

Furthermore, the study is susceptible to selection bias. Our cohort consisted exclusively of patients who had already achieved clinical remission on IV therapy before switching. This means our data may overrepresent patients with a more favourable treatment response profile. As our findings demonstrate success only in the context of clinical stability, it remains unclear whether SC IFX would yield similar outcomes in patients with active pouchitis.

Regarding generalisability, we must emphasise the specific context of our institution. As a specialised tertiary national referral centre, our patient population is characterised by high complexity and a high volume of pouch-related complications. While this provides a concentrated look at a rare condition, it introduces a referral bias that may not reflect the broader patient landscape of IPAA patients in secondary care settings. The outcomes reported here are products of a high-volume environment with significant multidisciplinary expertise in pouch management. Therefore, our results may not be fully generalisable to non-specialist units.

Ultimately, while this study provides valuable preliminary evidence for the transition to SC IFX in this niche population, prospective, multi-centre randomised controlled trials are required. Such studies should include larger, more diverse cohorts and comparative arms to validate these findings and establish definitive clinical protocols.

## 5. Conclusions

In conclusion, for clinically stable patients with chronic pouch conditions, switching from IV to SC IFX appears feasible. This approach might offer significant logistical and economic advantages to both the patient and the healthcare provider. These findings warrant confirmation in larger patient cohorts with longer-term follow-up.

## Figures and Tables

**Table 1:** Patient Baseline Characteristics.

Characteristic	N = 7
Age (years)	50.6 (36.3–67.5)
Age at UC diagnosis (years)	20.0 (13.0–26.0)
Ethnicity	
Caucasian	6/7 (85.7%)
South Asian	1/7 (14.3%)
Females	4/7 (57.1%)
Smoking	2/7 (28.6%)
PSC	0/7 (0.0%)
Colectomy indication	
Refractory disease	7/7 (100.0%)
Dysplasia	0/7 (0.0%)
Anti-TNF treatment prior to colectomy	
Yes	0/7 (0.0%)
No	7/7 (100.0%)
Cuffitis	2/7 (28.6%)
Fistulae	2/7 (28.6%)
Anti-TNF indication	
PPI + pouchitis	5/7 (71.4%)
Pouchitis	2/7 (28.6%)

## Data Availability

Dataset available on request from the authors.

## Supplementary Materials

The following supporting information can be downloaded at: https://www.mdpi.com/article/10.3390/jcm15052053/s1, Table S1: individual patient data.
